# Maximizing biohydrogen production from watermelon peels using *Clostridium butyricum* NE133: a statistical optimization approach with Plackett–Burman and Box–Behnken designs

**DOI:** 10.1186/s13068-025-02652-3

**Published:** 2025-05-14

**Authors:** Norhan Elerakey, Abdel-Hamied M. Rasmey, Youseef M. Mohammed, Akram A. Aboseidah, Heba Hawary

**Affiliations:** 1 Department of Botany and Microbiology, Faculty of Science, Suez University, P.O. Box 43221, Suez, Egypt; 2 Department of Botany and Microbiology, Faculty of Science, Damanhour University, Damanhour, 22516 Egypt

**Keywords:** Biohydrogen, Plackett–Burman (PB) design, Box–Behnken (BB) design, Watermelon peels, *Clostridium butyricum*, Response surface methodology (RSM)

## Abstract

**Background:**

Biohydrogen production from agricultural waste is a promising strategy to address climate change and energy challenges. This study aimed to optimize the process parameters for biohydrogen production from watermelon peels (WMP) by *Clostridium butyricum* NE133 using statistical optimization techniques. Initial screening of eight significant variables influencing hydrogen production including, initial pH, incubation temperature, WMP concentration, inoculum volume, yeast extract, tryptone, sodium acetate, and ammonium acetate concentration was conducted by a Plackett–Burman (PB) design.

**Results:**

The results showed that four variables including, initial pH (*P* < 0.001), WMP concentration (*P* < 0.001), sodium acetate (*P* = 0.023), and ammonium acetate (*P* = 0.048) had statistically significant effects on hydrogen production. The model curvature (*P* = 0.040) indicated that it was significant. Box–Behnken (BB) design under response surface methodology (RSM) was employed to optimize the four selected variables to maximize hydrogen production. The optimal conditions for maximizing hydrogen production from WMP by *C. butyricum* were: initial pH of 8.98, WMP concentration of 44.75%, sodium acetate 4.49 gL^−1^, and ammonium acetate 1.15 gL^−1^ at with predicted H_max_ of 4703.23 mLL^−1^. The determination coefficient R^2^ of the model was 0.9902 with the lack of fit F-value was 1.86.

**Conclusions:**

The confirmation experiment revealed only a 0.59% difference between the predicted and experimental hydrogen production, indicating that the optimum conditions were actual with the least error. Improvement of about 103.25% in hydrogen production from WMP by *C. butyricum* NE133 was achieved after the optimization process.

**Graphical Abstract:**

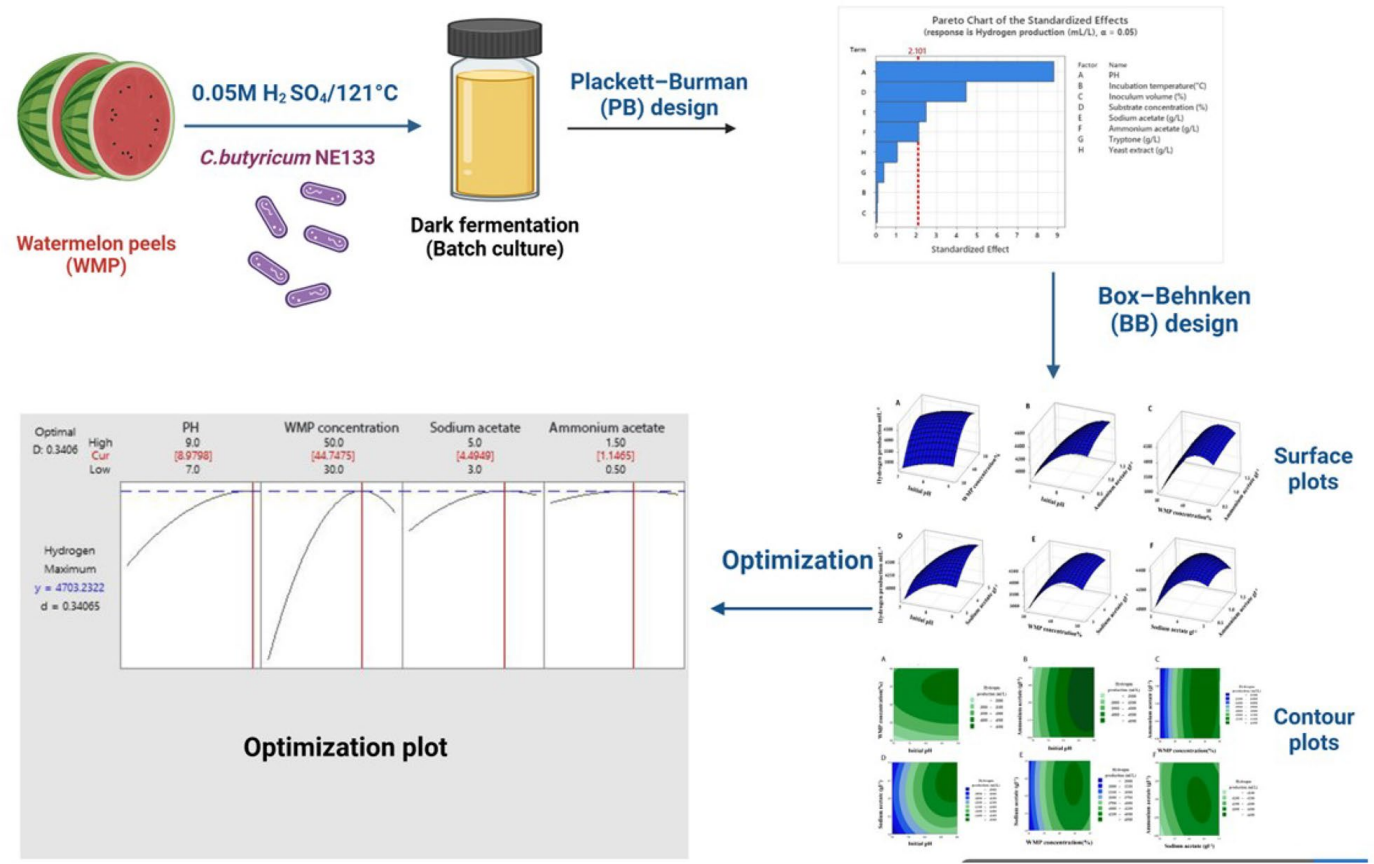

**Supplementary Information:**

The online version contains supplementary material available at 10.1186/s13068-025-02652-3.

## Background

One of the major issues around the world is the depletion of fossil fuels, which has caused an imbalance between energy supply and demand, leading to the global energy crisis. This has prompted the search for a more sustainable and cleaner energy source for current and future generations. Biomass-based fuel is one of the promising alternatives to fossil fuels due to its numerous advantages, such as its renewable nature, lower greenhouse gas emissions, and widely distributed supply around the world [1, 2].

Biohydrogen is a well-recognized biomass-based fuel with the highest energy content (143 kJ g^−1^) and is carbon neutral compared to other hydrocarbon fuels [3]. Hydrogen can be produced through biological, chemical, and physical processes. The biological method of hydrogen production has garnered more attention than chemical and physical methods because it uses less energy and is sustainable. There are two types of biological hydrogen production: dark fermentation by fermentative anaerobic bacteria and photo-fermentation process by photosynthetic bacteria and algae. Dark fermentation (DF) technology is the most efficient biological route for hydrogen production. Unlike photo-fermentation, DF can continuously produce hydrogen from several feedstocks without external energy input [4]. Several types of bacteria, including *Enterobacter*, *Bacillus*, and *Clostridium*, are known to be involved in the DF process [5].

Biohydrogen can be used in various applications across various sectors including electricity generation, construction, industrial processes, and transportation. It can be applied in chemical industries like ammonia and methanol synthesis, refineries, and steel manufacturing. These applications contribute to reducing reliance on fossil fuels in the industrial sector. Additionally, the possible utilization of hydrogen fuel cells in electric vehicles, buses, trains, and ships provides advantages such as quick refueling times, excellent driving ranges, and the generation of electrical energy with zero carbon dioxide emissions [6].

Watermelon (*Citrullus lanatus*) is a plant belonging to the Cucurbitaceae family and Citrullus genus [7]. Watermelon fruits biomass can be divided into three major parts: peel, seed, and flesh. The flesh of watermelon fruits makes up about 40% of their weight, while its peel and seeds make up roughly 60% of the fruit. This is a significant percentage of agricultural waste when compared to the peels of similar fruits, such as melon (which makes up 40%), pumpkin (45%), musk melon (35%), papaya (47%), bitter apple (30%) [8, 9]. About 50% of the watermelon is edible, while the other half is typically discarded. According to watermelon production in 2017–2018, approximately 42 million tonnes of watermelon by-products (rind and seeds) were produced during the preparation and consumption of watermelon by homemade fruit juice producers, fruit juice processing industries, and restaurants. These residue waste materials are carelessly thrown into the environment without any useful recycling, creating environmental problems [10].

Watermelon fruit is a rich source of vitamins (A, B, C, and E), free amino acids (arginine and citrulline), carotenoids (primarily lycopene), mineral salts (K, Mg, Ca, and Fe), and phenolic compounds [11]. Yargamji et al. [12] revealed that watermelon rinds have a carbohydrate content of 72.42% which can be fermented to produce sugars for biohydrogen production.

Environmental factors including temperature, pH, inoculum size, tryptone, yeast extract, sodium acetate, ammonium acetate, and substrate concentration have a significant impact on the production of biohydrogen through DF [13, 14]. To maximize hydrogen production, these factors must be optimized. However, carrying out the optimization using a traditional method, sometimes referred to as “one factor at a time”, is tedious and time-consuming [15]. Plackett–Burman’s (PB) statistical design and response surface methodology (RSM) can overcome this limitation. They are useful tools for screening, identifying, and optimizing the most significant elements from an extensive list of potential interfering factors [16]. These statistical methods save time, show the interactive effects between tested variables, and reduce errors in parameter determination [17].

This study presents a novel and systematic approach for enhancing biohydrogen production from watermelon peels (WMP) as a sustainable substrate using *Clostridium butyricum* NE133. Unlike previous studies, which have largely focused on other agricultural wastes, this is the first study to apply an integrated statistical strategy for optimizing hydrogen yield from WMP. The current investigation aimed to screen the most significant variables influencing hydrogen production from WMP by *Clostridium butyricum* NE133 using a PB design. Moreover, the levels of the screened factors were optimized by the Box–Behnken (BB) design of RSM to provide the optimal conditions for enhancing the hydrogen yield from WMP. Additionally, this study incorporated the kinetic modeling using the Modified Gompertz Model (MGM) to validate the optimization strategy and provide a deeper understanding of the fermentation process dynamics.

## Material and methods

### Preparation of feedstock

Watermelon (*Citrullus lanatus*) peels were collected from local fruit shops, chopped into small pieces using sterile knives, and blended with sterilized distilled water to facilitate uniform homogenization and prepare a suitable fermentation medium. The physicochemical composition of WMP (per 100 g) is described in Table S1 (supplementary file).

The blended WMP hydrolysate was treated with sulfuric acid (0.05M) for 20 min at 121 °C. Then, the initial pH of the treated WMP hydrolysate was adjusted individually to the desired pH (according to the pH values of the statistical design) using NaOH 0.1 N and HCl 0.1 N before being used as a substrate for hydrogen production.

### Bacterial isolate source

The hydrogen-producing bacterial isolate *Clostridium butyricum* NE133 (accession number: PP581793) isolated in our previous study [18], was used for the fermentation processes.

### Inoculum preparation

One mL of 48-h-old culture was added into 100 mL of sterilized Reinforced *Clostridium* broth media (RCM) to prepare bacterial inoculum (3 × 10^6^ cells mL^−1^). The RCM media consists of the following (gL^−1^): peptone 10.0 g, beef extract 10.0 g, yeast extract 3.0 g, glucose 5.0 g, sodium chloride 5.0 g, sodium acetate 3.0 g, starch 1.0 g, and l-cysteine-HCL 0.5 g [19]. The RCM broths were then incubated under anaerobic conditions at 37 °C for 48 h (h) before being used as the inoculum in the batch experiment.

### Experimental setup for batch assays for hydrogen production

The hydrogen production batch assays were conducted in 125 mL serum bottles with 90 mL working volume. The WMP medium was composed of WMP hydrolysate (treated with 0.05 M H_2_SO_4_/121 °C) along with different levels and concentrations of variables based on the design trials. The serum bottles were flushed with nitrogen gas to create anaerobic conditions and then capped with a rubber stopper. The evolved gas mixture will be passed through a 2 M NaOH solution to absorb the most carbon dioxide produced. The bottles were then incubated at different temperatures according to the design for 96 h. At each time interval, the total volume of the gas was measured in inverted cylinders using the water displacement method (Fig. S2, supplementary file) [20].

### Screening and identifying the significant factors for hydrogen production using PB design

Eight different variables were screened to identify the significant factors affecting hydrogen production by *C. butyricum* NE133 using PB design [21]. The screened factors had two levels, namely, X_1_ (initial pH), X_2_ (incubation temperature), X_3_ (inoculum volume), X_4_ (WMP concentration), X_5_ (sodium acetate), X_6_ (ammonium acetate), X_7_ (tryptone), and X_8_ (yeast extract). The levels of each factor are listed in Table 1 with the coded and uncoded form of high (+ 1) and low (− 1) values, whereas Table 2 represents the experimental PB design matrix using the statistical software Minitab 21.

**Table 1:** Actual and coded values for the screened variables at two levels using the PB design for hydrogen production

Factors	Symbol code	Actual values of coded factors	
		(− 1)	(1)
Initial pH	X_1_	5	8
Incubation temperature (°C)	X_2_	28	37
Inoculum volume (%)	X_3_	5	25
Watermelon peel (WMP) concentration (%)	X_4_	10	40
Sodium acetate (gL^−1^)	X_5_	1	4
Ammonium acetate (gL^−1^)	X_6_	1	5
Tryptone (gL^−1^)	X_7_	1	7
Yeast extract (gL^−1^)	X_8_	0.5	3

**Table 2:** PB design matrix for screening independent variables with actual and coded values influencing hydrogen production

Trial	Variables								Response	
	X_1_	X_2_	X_3_	X_4_	X_5_	X_6_	X_7_	X_8_	Hydrogen production (mLL^−1^)	
	Initial pH	Incubation temperature (°C)	Inoculum volume (%)	WMP (%)	Sodium acetate (gL^−1^)	Ammonium acetate (gL^−1^)	Tryptone (gL^−1^)	Yeast extract (gL^−1^)	Experimental	Predicted
1	1 (8)	− 1 (28)	− 1 (5)	− 1 (10)	− 1 (1)	1 (5)	− 1 (1)	1 (3)	827.00	1136.22
2	1 (8)	1 (37)	− 1 (5)	− 1 (10)	− 1 (1)	− 1 (1)	1 (7)	− 1 (0.5)	1171.67	1734.44
3	1 (8)	1 (37)	1 (25)	− 1 (10)	− 1 (1)	− 1 (1)	− 1 (1)	1 (3)	1865.00	1610.78
4	1 (8)	1 (37)	1 (25)	1 (40)	− 1 (1)	− 1 (1)	− 1 (1)	− 1 (0.5)	2630.00	2863.39
5	1 (8)	1 (37)	1 (25)	1 (40)	1 (4)	− 1 (1)	− 1 (1)	− 1 (0.5)	3280.33	3423.33
6	− 1 (5)	1 (37)	1 (25)	1 (40)	1 (4)	1 (5)	− 1 (1)	− 1 (0.5)	265.67	353.06
7	1 (8)	− 1 (28)	1 (25)	1 (40)	1 (4)	1 (5)	1 (7)	− 1 (0.5)	3607.67	2874.17
8	− 1 (5)	1 (37)	− 1 (5)	1 (40)	1 (4)	1 (5)	1 (7)	1 (3)	160.33	297.72
9	1 (8)	− 1 (28)	1 (25)	− 1 (10)	1 (4)	1 (5)	1 (7)	1 (3)	995.33	1221.56
10	1 (8)	1 (37)	− 1 (5)	1 (40)	− 1 (1)	1 (5)	1 (7)	1 (3)	2652.67	2028.61
11	− 1 (5)	1 (37)	1 (25)	− 1 (10)	1 (4)	− 1 (1)	1 (7)	1 (3)	255.00	184.67
12	− 1 (5)	− 1 (28)	1 (25)	1 (40)	− 1 (1)	1 (5)	− 1 (1)	1 (3)	245.00	179.11
13	1 (8)	− 1 (28)	− 1 (5)	1 (40)	1 (4)	− 1 (1)	1 (7)	− 1 (0.5)	3512.00	3333
14	1 (8)	1 (37)	− 1 (5)	− 1 (10)	1 (4)	1 (5)	− 1 (1)	1 (3)	1701.33	1670.67
15	− 1 (5)	1 (37)	1 (25)	− 1 (10)	− 1 (1)	1 (5)	1 (7)	− 1 (0.5)	210.67	183.33
16	− 1 (5)	− 1 (28)	1 (25)	1 (40)	− 1 (1)	− 1 (1)	1 (7)	1 (3)	395.00	463.33
17	1 (8)	− 1 (28)	− 1 (5)	1 (40)	1 (4)	− 1 (1)	− 1 (1)	1 (3)	3680.00	3188.72
18	− 1 (5)	1 (37)	− 1 (5)	− 1 (10)	1 (4)	1 (5)	− 1 (1)	− 1 (0.5)	220.00	110.67
19	1 (8)	− 1 (28)	1 (25)	− 1 (10)	− 1 (1)	1 (5)	1 (7)	− 1 (0.5)	863.00	1301.11
20	− 1 (5)	1 (37)	− 1 (5)	1 (40)	− 1 (1)	− 1 (1)	1 (7)	1 (3)	275.33	230.51
21	− 1 (5)	− 1 (28)	1 (25)	− 1 (10)	1 (4)	− 1 (1)	− 1 (1)	1 (3)	352.00	205.39
22	− 1 (5)	− 1 (28)	− 1 (5)	1 (40)	− 1 (1)	1 (5)	− 1 (1)	− 1 (0.5)	215.67	398
23	− 1 (5)	− 1 (28)	− 1 (5)	− 1 (10)	1 (4)	− 1 (1)	1 (7)	− 1 (0.5)	171.00	229.06
24	− 1 (5)	− 1 (28)	− 1 (5)	− 1 (10)	− 1 (1)	− 1 (1)	− 1 (1)	− 1 (0.5)	130.33	84.67
25	0 (6.5)	0 (32)	0 (15)	0 (25)	0 (2.5)	0 (3)	0 (4)	0 (1.5)	2055.33	1825.46
26	0 (6.5)	0 (32)	0 (15)	0 (25)	0 (2.5)	0 (3)	0 (4)	0 (1.5)	1898.67	1825.46
27	0 (6.5)	0 (32)	0 (15)	0 (25)	0 (2.5)	0 (3)	0 (4)	0 (1.5)	1800.33	1825.46

The range of values specified for each variable at both the upper and lower levels of the PB factorial design was derived from studies that assessed hydrogen production through fermentation processes utilizing various lignocellulosic substrates [22, 23]. The eight assigned factors were screened in 27 experimental runs (24 experimental runs and 3 center points experiments) and the hydrogen production (mLL^−1^) was evaluated as the response. The main effect of each variable was determined according to the subsequent equation (Eq. 1):

1 $${E}_{{x}_{i}}=\frac{2\left(\Sigma {M}_{i+}-{M}_{{i}_{-}}\right)}{N},$$

where *E*_*Xi*_ is the effect of the tested variable, *M*_*i*+,_ and *M*_*i*-_ are hydrogen production in trials where the independent variable (*X*_*i*_) measured was present at the high and low concentration, respectively, and N is the total number of runs. To determine variable significance, statistical t-values for equal unpaired samples were calculated concerning observations. Variables with a 95% level of significance (*P* < 0.05) were identified as having a significant impact on hydrogen production and were selected for further optimization.

### Optimization of hydrogen production by *C. butyricum* NE133 using RSM

A four-variable Box–Behnken (BB) design under RSM was applied to optimize the significant variables that enhanced biohydrogen production [24]. The center points and parameters were selected according to the PB design. In this model, the most significant independent variables, named initial pH (X_1_), WMP concentration (X_4),_ sodium acetate (X_5_), and ammonium acetate (X_6_) are included and each factor can be examined at three different levels, low (−), high (+) and basal (0) as shown in Table 3. Twenty-seven trials and their observations (shown in the results section) were fitted to the following second-order polynomial model (Eq. 2):

2 $$\text{Y}={\text{b}}_{0}+{\text{b}}_{1}{\text{x}}_{1}+{\text{b}}_{2}{\text{x}}_{2}+{\text{b}}_{3}{\text{x}}_{3}+{\text{b}}_{4}{\text{x}}_{4}+{\text{b}}_{12}{\text{x}}_{1}{\text{x}}_{2}+{\text{b}}_{13}{\text{x}}_{1}{\text{x}}_{3}+{\text{b}}_{14}{\text{x}}_{1}{\text{x}}_{4}+{\text{b}}_{23}{\text{x}}_{2}{\text{x}}_{3}+{\text{b}}_{24}{\text{x}}_{2}{\text{x}}_{4}+{\text{b}}_{34}{\text{x}}_{3}{\text{x}}_{4}+{\text{b}}_{11}{\text{x}}_{1}^{2}+{\text{b}}_{22}{\text{x}}_{2}^{2}{+\text{b}}_{33}{\text{x}}_{3}^{2}{+\text{b}}_{44}{\text{x}}_{4}^{2},$$

where Y is the dependent variable (hydrogen production); X_1_, X_2_, X_3_ and X_4_ are the independent variables; b_o_ is the regression coefficient at the center point; b_1_, b_2_, b_3,_ and b_4_ are linear coefficients; b_12_, b_13_, b_14_, b_23_, b_24,_ and b_34_ are second-order interaction coefficients; and b_11_, b_22_, b_33,_ and b_44_ are squared coefficients. The quality of the fit of the polynomial model equation was expressed by the coefficient of determination (R^2^). The values of the coefficients were calculated and the optimum concentrations were predicted using Minitab 21 software.

**Table 3:** BB experimental design with actual and coded operational variables and response for optimization of hydrogen production by *C. butyricum* NE133

Trials	Initial pH (X_1_)	WMP concentration (%) (X_4_)	Sodium acetate (gL^−1^) (X_5_)	Ammonium acetate (gL^−1^) (X_6_)	Hydrogen production (mLL^−1^)	
					Experimental	Predicted
1	− 1 (7)	− 1 (30)	0 (4)	0 (1)	2792.333333	2738.39
2	1 (9)	− 1 (30)	0 (4)	0 (1)	3219	3265.44
3	− 1 (7)	1 (50)	0 (4)	0 (1)	3935.33	3922
4	1 (9)	1 (50)	0 (4)	0 (1)	4375	4462.05
5	0 (8)	0 (40)	− 1 (3)	− 1 (0.5)	3946	4031.83
6	0 (8)	0 (40)	1 (5)	− 1 (0.5)	4288.67	4334.28
7	0 (8)	0 (40)	− 1 (3)	1 (1.5)	4185	4172.50
8	0 (8)	0 (40)	1 (5)	1 (1.5)	4306.33	4253.61
9	− 1 (7)	0 (40)	0 (4)	− 1 (0.5)	3888.33	3942.86
10	1 (9)	0 (40)	0 (4)	− 1 (0.5)	4444.67	4403.75
11	− 1 (7)	0 (40)	0 (4)	1 (1.5)	3900	3900.20
12	1 (9)	0 (40)	0 (4)	1 (1.5)	4601.67	4506.42
13	0 (8)	− 1 (30)	− 1 (3)	0 (1)	2968	2925.36
14	0 (8)	1 (50)	− 1 (3)	0 (1)	4123.67	4096.30
15	0 (8)	− 1 (30)	1 (5)	0 (1)	3111.33	3097.97
16	0 (8)	1 (50)	1 (5)	0 (1)	4305.33	4307.25
17	− 1 (7)	0 (40)	− 1 (3)	0 (1)	3801	3801.80
18	1 (9)	0 (40)	− 1 (3)	0 (1)	4210.33	4206.20
19	− 1 (7)	0 (40)	1 (5)	0 (1)	3852.67	3864.42
20	1 (9)	0 (40)	1 (5)	0 (1)	4520.33	4527.14
21	0 (8)	− 1 (30)	0 (4)	− 1 (0.5)	3155	3110.42
22	0 (8)	1 (50)	0 (4)	− 1 (0.5)	4350.33	4249.86
23	0 (8)	− 1 (30)	0 (4)	1 (1.5)	2981.67	3089.75
24	0 (8)	1 (50)	0 (4)	1 (1.5)	4278.33	4330.53
25	0 (8)	0 (40)	0 (4)	0 (1)	4471	4407.11
26	0 (8)	0 (40)	0 (4)	0 (1)	4350.33	4407.11
27	0 (8)	0 (40)	0 (4)	0 (1)	4400	4407.11

### Analytical methods

To monitor and collect data during fermentation tests, analyses were performed in the broth media of the batch cultures. Gas chromatography–mass spectrometry (GC–MS) analysis is used to determine fatty acid esters (FAE) of volatile fatty acids (VFAs) in spent fermentation media. GC–MS analysis of WMP fermentation media extract was performed before and after fermentation by *C. butyricum* NE133 under optimal conditions for hydrogen production. The GC–MS analysis was performed using a Thermo Scientific, Trace GC Ultra/ISQ Single Quadrupole MS, TG-5MS fused silica capillary column (30 m, 0.251 mm, 0.1 mm film thickness). An electron ionization system with ionization energy of 70 eV was used for GC–MS detection. At a constant flow rate of 1 mL min^−1^, helium gas was used as a carrier gas. Also, the injector and MS transfer line temperature was programmed at 280 °C. The oven temperature was initially set at 45 °C (hold 2 min) to 150 °C at an increasing rate of 7 °C min^−1^, then programmed to 270 °C at an increasing rate 5 °C min^−1^ (hold 2 min) and finally programmed to 310 °C for 10 min at an increasing rate of 3.5 °C min^−1^. A percent relative peak area served as the basis for quantifying all the identified components. The relative retention time and mass spectra of the identified compounds were compared with those of the NIST, WILLY library data of the GC/MS system.

### Kinetic modeling

A kinetic analysis was used to evaluate the hydrogen production dynamics by *C. butyricum NE133* over 96 h at 12-h intervals. The produced cumulative hydrogen curves were fitted using a modified Gompertz model (MGM) (Eq. 3) to estimate the lag phase λ (the time to start producing hydrogen), hydrogen production potential (H), and hydrogen production rate (R). The hydrogen production rate ${R}_{{H}_{2}}$(mLL^−1^h^−1^) at time t (h) was measured using (Eq. 4). The t_95_ (time at which 95% of maximal cumulative hydrogen was generated) was calculated using (Eq. 5) [25]:

3 $${\text{H}}_{\left(\text{C}\right)}={\text{H}}_{\text{max }}.\text{exp}\left\{-\text{exp}\left[\frac{{\text{R}}_{\text{max}}.\text{e}}{{\text{H}}_{\text{max}}}\left(\uplambda -\text{t}\right)+1\right]\right\},$$

4 $${R}_{{H}_{2}}={R}_{\text{max} }.\text{exp}\left\{\left[\frac{{R}_{max}.e}{{H}_{max}}\left(\lambda -t\right)+1\right]+\left[\frac{{R}_{\text{max}}.e}{{H}_{\text{max}}}\left(\lambda -t\right)+1\right]+1\right\},$$

5 $${t}_{95}=\frac{{H}_{\text{max}}}{{R}_{\text{max}}.e}\left[1-\text{ln}(-\text{ln}0.95)\right]+\lambda ,$$

where: 

H(c) = the cumulative HP (mLL^−1^)

R_max_ = the maximum rate of HP (mLL^−1^h^−1^)

e = 2.718281

λ = lag phase time (h).

## Results

### Screening of significant variables for hydrogen production by *C. butyricum* NE133

**Fig. 1:**
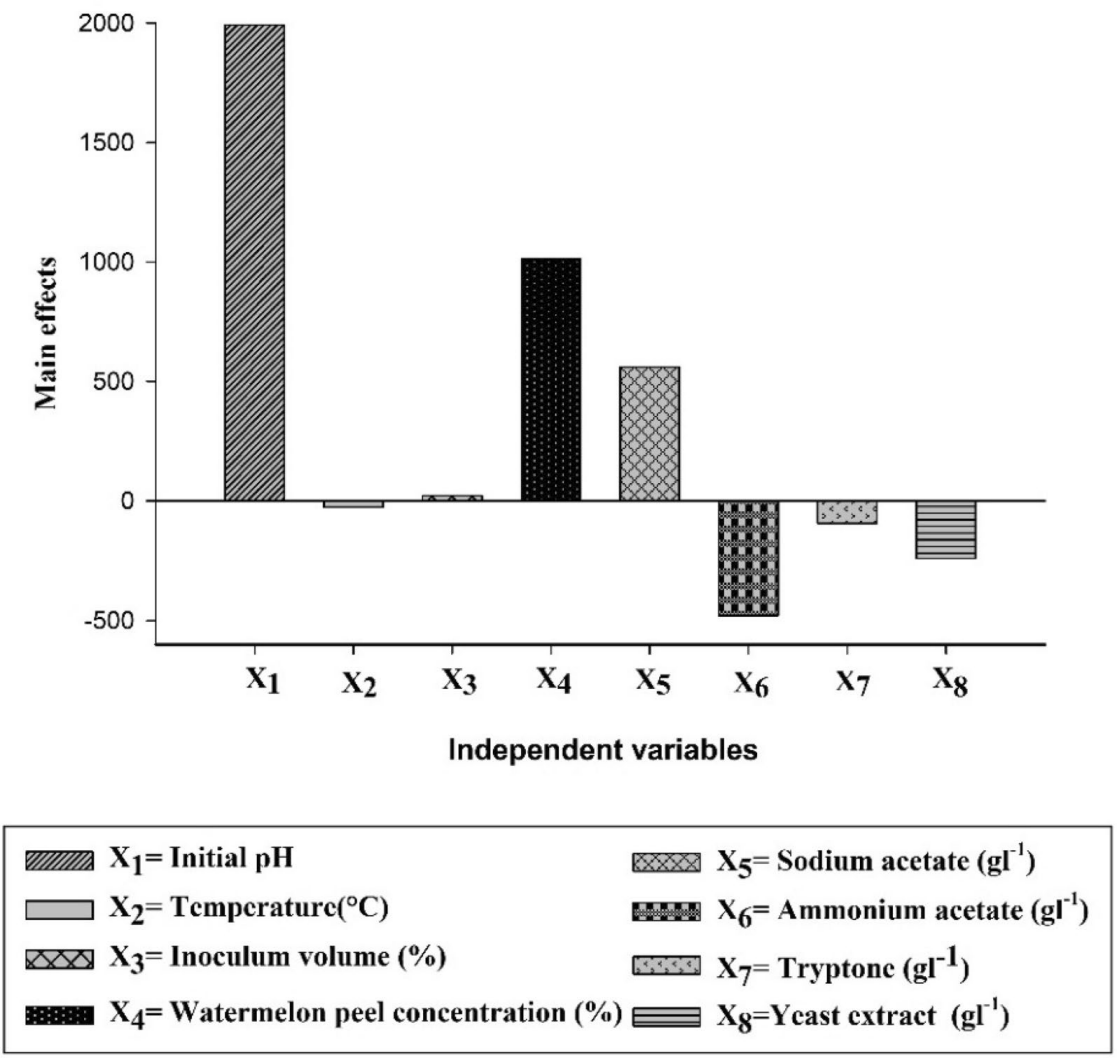
Main effects of independent variables examined in PB design for hydrogen production response

**Fig. 2:**
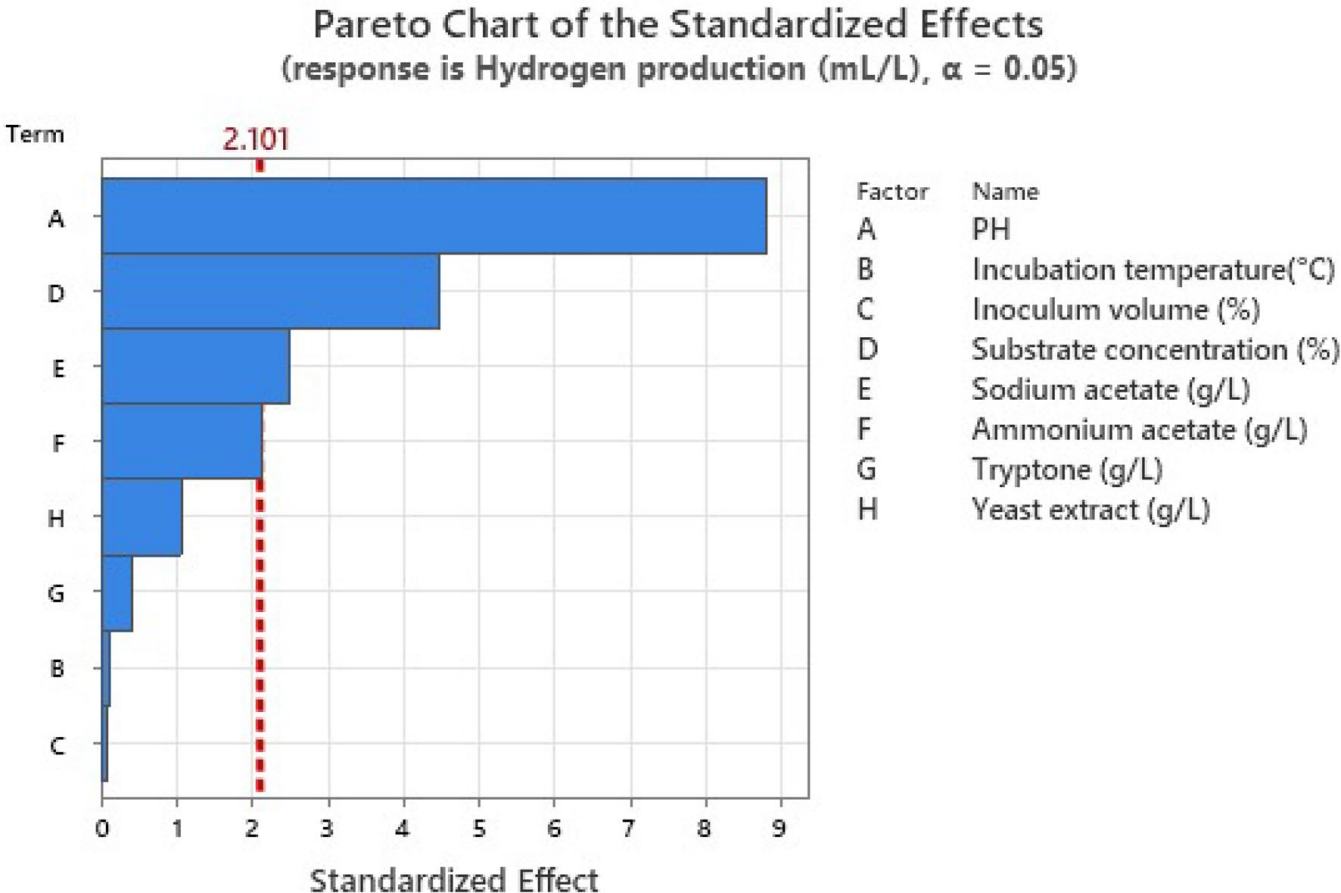
Pareto chart of the standardized effects of hydrogen production (mLL^−1^) examined in PB design (Alpha = 0.05)

The effect of the variables on hydrogen production was determined by conducting 24 experiments given by the PB factorial design model (Table 2). All 27 runs in PB factorial design successfully produced hydrogen gas through dark fermentation. The maximum production of hydrogen from WMP (3680 mLL^−1^) was revealed on trial (17) at which four factors obtained a positive effect; initial pH, WMP concentration, sodium acetate, and yeast extract, while the other four factors; incubation temperature, inoculum volume, tryptone, and ammonium acetate showed a negative effect. The main effect of each variable was calculated as the difference between the average measurements of that factor at the low level (− 1) and high level (+ 1). The results of the main effect are presented in Table 4. The tested variable’s effect E_Xi_ has a positive sign when the influence on hydrogen production is higher at a high level and a negative sign when it is greater at a low level (Fig. 1). The results designated that the presence of high levels of X_1_ (initial pH), X_3_ (inoculum volume), X_4_ (WMP concentration), and X_5_ (sodium acetate concentration) in the fermentation medium positively affected hydrogen production. These results are supported by the Pareto chart shown in Fig. 2, which shows higher effects in the upper portion and then progresses down to lower effects. The F-value of the model (13.77) implies that it was significant. The accuracy of determination coefficient R^2^ (85.95%) reveals the adequacy of the experimental model. The model curvature *P*-value was 0.040 indicating a significant difference between center point experiments and the average output responses. The lack of fit *P*-value was 0.057. It was estimated from Fig. 2 that the initial pH (*P* < 0.001) and WMP concentration (*P* < 0.001) were the most significant variables that affected hydrogen production, followed by sodium acetate (*P* = 0.023) and ammonium acetate (*P* = 0.048), respectively. Therefore, these four significant variables were selected for the following optimization step. Variables with insignificant effects were excluded from the next optimization experiment. However, they were still used in all trials at their (− 1) or (+ 1) levels, depending on their negative or positive effect on hydrogen production.

**Table 4:** Estimated effect, regression coefficient, and corresponding F and *P* values for hydrogen production in the PB design

Source	Effect	Coef	F-value	*P*-value
Model	–	–	13.77	0.000
Linear	–	–	13.77	0.000
Initial pH	1991	995	77.95	0.000
Incubation temperature (°C)	− 26	− 13	0.01	0.911
Inoculum volume (%)	21	10	0.01	0.928
Substrate concentration (%)	1013	507	20.19	0.000
Sodium acetate (gL^−1^)	560	280	6.17	0.023
Ammonium acetate (gL^−1^)	− 479	− 240	4.52	0.048
Tryptone (gL^−1^)	− 95	− 48	0.18	0.678
Yeast extract (gL^−1^)	− 240	− 120	1.13	0.302
Curvature	–	–	4.95	0.040
Lack-of-fit	–	–	17.01	0.057

### Optimization of hydrogen production using response surface methodology (RSM)

#### Modeling of hydrogen production by Box–Behnken (BB) design of RSM

Four significant independent variables: initial pH (X_1_), WMP concentration (X_4_), sodium acetate (X_5_), and ammonium acetate (X_6_), suggested by the PB design, were further investigated. Each variable was tested at three levels (− 1, 0, and + 1) according to the BB design. However, temperature (28) °C, inoculum volume (25) %, tryptone (1) gL^−1^, and yeast extract (0.5) gL^−1^ were treated as constant factors. Table 3 represents the design matrix of the variables together with the experimental results of hydrogen production. Experiments from 1 to 27 were conducted at various combinations, while experiments from 25 to 27 were performed under the same conditions. As shown in Table 3, the highest hydrogen production recorded (4601.67 mLL^−1^) was achieved by trial number 12. On the other hand, the lowest hydrogen production (2792.33 mLL^−1^) was achieved by trial number 1. To accurately predict the optimal point, a second-order polynomial function was fitted to the hydrogen production results of the applied BB experiment. The results were obtained using the Minitab 21 software, yielding a coefficient of determination R^2^ value of 0.9902. According to the results of the statistical analysis, the relationship between the response (hydrogen production) and the four independent variables can be represented by the following equation (Eq. 6):

6 $$\text{Y}=4407.1+266.8 {\text{X}}_{1}+595.1 {\text{X}}_{4}+95.9 {\text{X}}_{5}+15.0 {\text{X}}_{6}-158.5 {\text{X}}_{1}^{2}-651.7{\text{X}}_{4}^{2}- 148.7 {\text{X}}_{5}^{2}-60.3 {\text{X}}_{6}^{2}+3.3 {\text{X}}_{1}{\text{X}}_{4}+ 64.6 {\text{X}}_{1}{\text{X}}_{5}+36.3 {\text{X}}_{1}{\text{X}}_{6}+9.6 {\text{X}}_{4}{\text{X}}_{5}+25.3 {\text{X}}_{4}{\text{X}}_{6} -55.3 {\text{X}}_{5}{\text{X}}_{6},$$

where Y is predicted hydrogen production (dependent variable); X_1_, X_4_, X_5_ and X_6_ are coded values of initial pH, WMP concentration, sodium acetate, and ammonium acetate, respectively. The F-value of the model was determined to be 86.85 with a *P*-value less than 0.001. The determination coefficient R^2^ was 0.9902. Also, the predictive R-square was 94.70%, the adjusted R-squared was 97.88%, and the lack of fit F-value was 1.86 with a *P*-value of 0.40 (Table 5).

**Table 5:** Analysis of variance (ANOVA) and results of regression analysis of BB design for hydrogen production

Source	DF	Adj SS	Adj MS	F-value	*P*-value	Significance
Model	14	7,672,586	548,042	86.85	0.000	HS*
Linear	4	5,216,163	1,304,041	206.65	0.000	HS
X_1_	1	854,046	854,046	135.34	0.000	HS
X_4_	1	4,249,081	4,249,081	673.36	0.000	HS
X_5_	1	110,335	110,335	17.49	0.001	HS
X_6_	1	2700	2700	0.43	0.525	NS^**^
Square	4	2,419,235	604,809	95.85	0.000	HS
X_1_*X_1_	1	133,961	133,961	21.23	0.001	HS
X_4_*X_4_	1	2,264,809	2,264,809	358.91	0.000	HS
X_5_*X_5_	1	117,986	117,986	18.7	0.001	HS
X_6_*X_6_	1	19,404	19,404	3.08	0.105	NS
2-Way interaction	6	37,188	6198	0.98	0.478	NS
X_1_*X_4_	1	42	42	0.01	0.936	NS
X_1_*X_5_	1	16,684	16,684	2.64	0.130	NS
X_1_*X_6_	1	5280	5280	0.84	0.378	NS
X_4_*X_5_	1	367	367	0.06	0.813	NS
X_4_*X_6_	1	2567	2567	0.41	0.536	NS
X_5_*X_6_	1	12,248	12,248	1.94	0.189	NS
Residual error	12	75,723	6310			
Lack-of-fit	10	68,366	6837	1.86	0.400	NS
Pure error	2	7356	3678			
Total	26	7,748,308				

*HS: Highly significant; **NS: Non significant

**Fig. 3:**
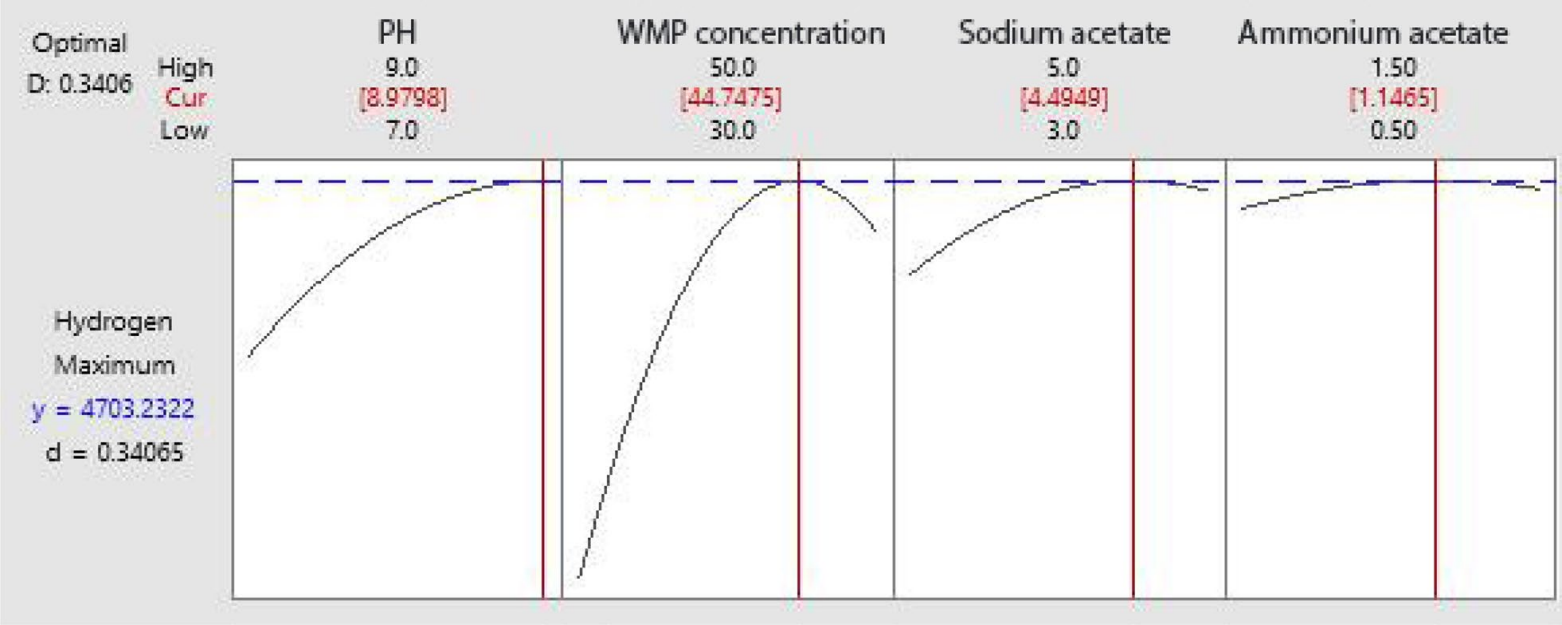
Optimization plot of hydrogen production by *C. butyricum* using BB design

**Fig. 4:**
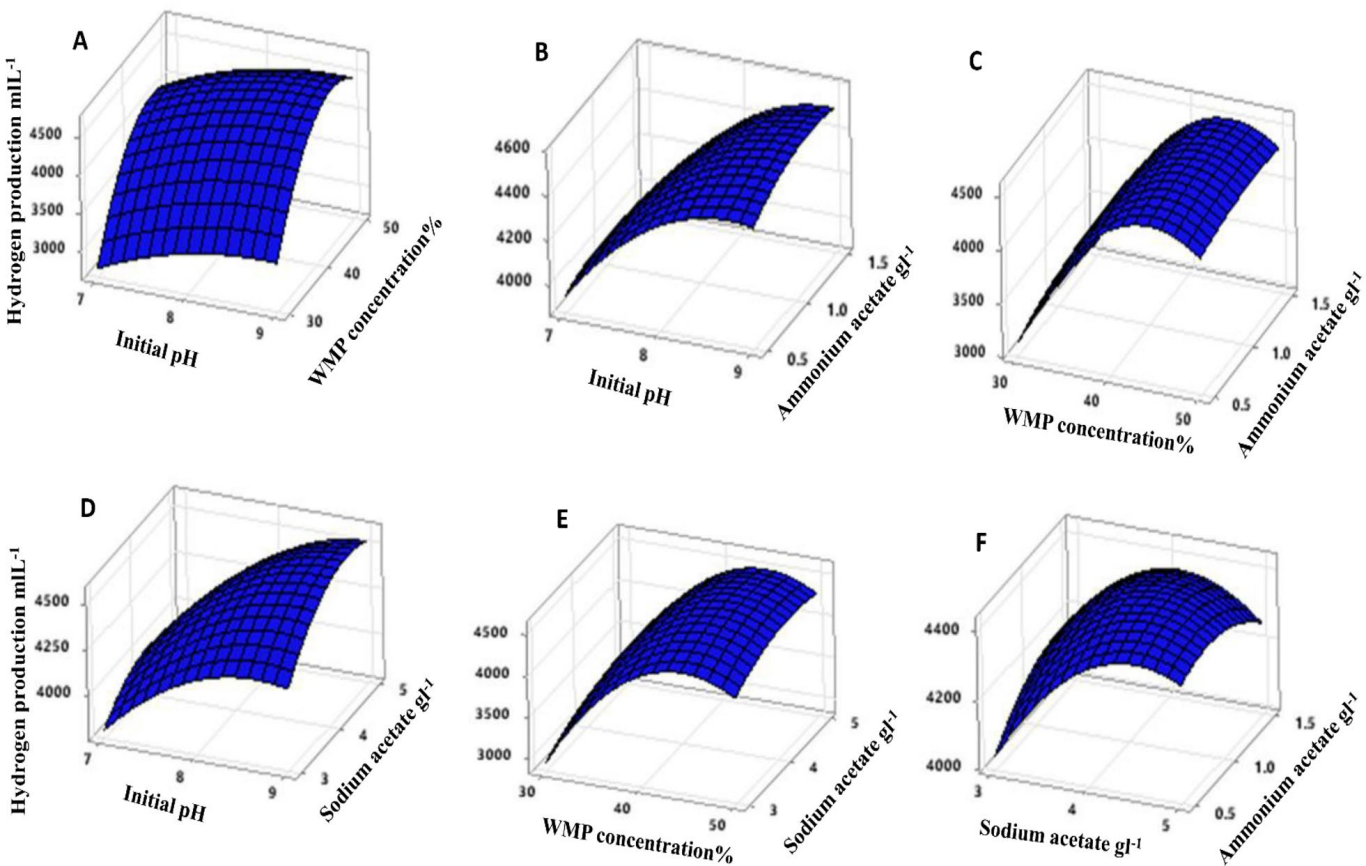
Response surface plots of hydrogen production by *C. butyricum* using independent variables: **A** initial pH and WMP concentration, **B** initial pH and ammonium acetate, **C** WMP concentration and ammonium acetate, **D** initial pH and sodium acetate, **E** WMP concentration and sodium acetate, **F** sodium acetate and ammonium acetate

**Fig. 5:**
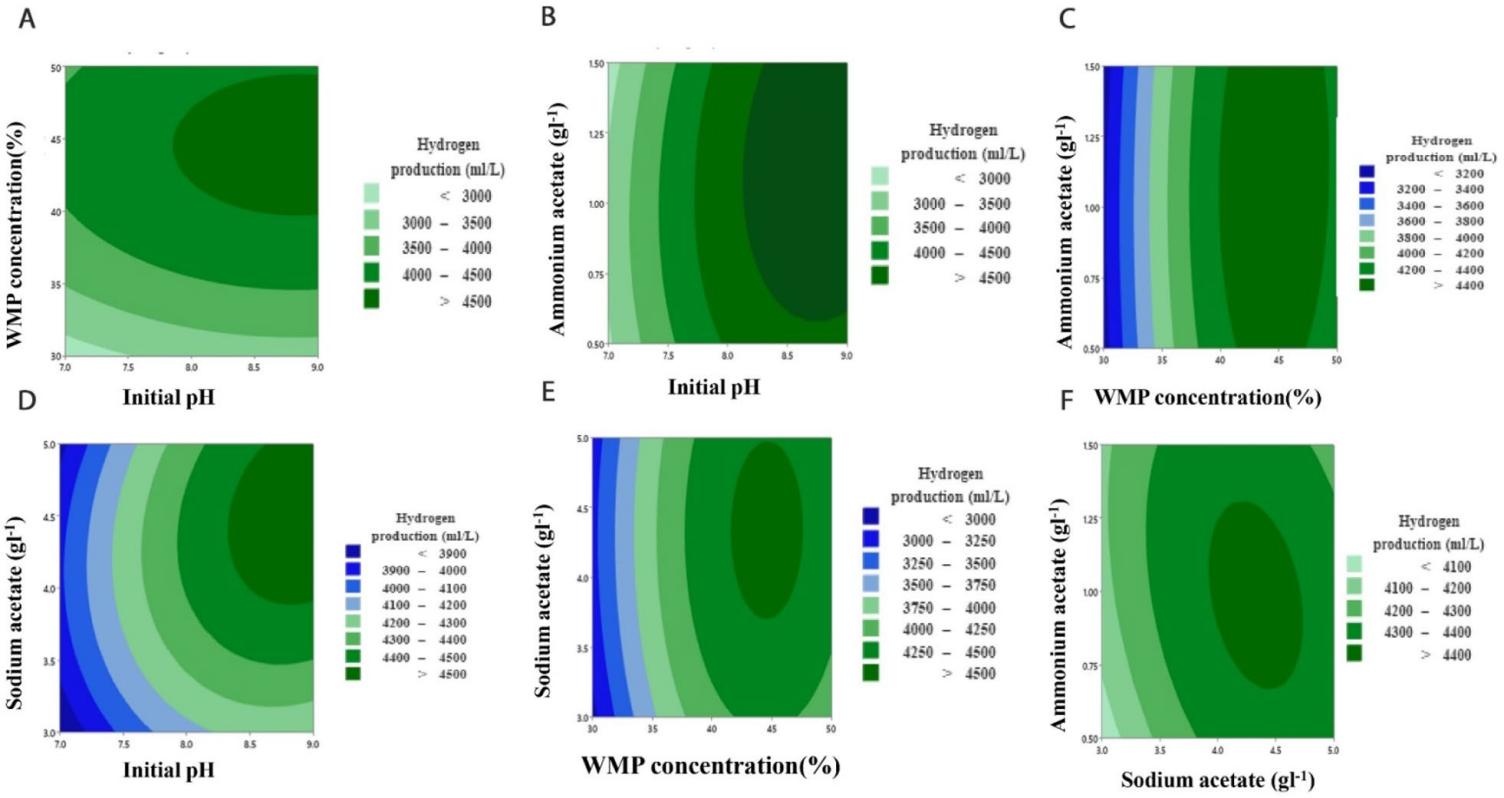
Contour plot of hydrogen production (mLL^−1^) production against: **A** initial pH and WMP concentration, **B** initial pH and ammonium acetate, **C** WMP concentration and ammonium acetate, **D** initial pH and sodium acetate, **E** WMP concentration and sodium acetate, **F** sodium acetate and ammonium acetate

Based on the parameters estimated and corresponding *P*-values, the linear and square terms of initial pH (X_1_), WMP concentration (X_4_), and sodium acetate (X_5_) had a significant effect on hydrogen production, with low *P*-values of less than 0.05. In contrast, the linear and square terms of ammonium acetate were found to be insignificant to hydrogen production. Equation (4) was set to zero concerning the corresponding variables to obtain the optimal condition for maximizing hydrogen production. It was found that the optimal values for H_max_ were initial pH 8.98, WMP concentration 44.75%, sodium acetate 4.49 gL^−1^, and ammonium acetate 1.15 gL^−1^. At these optimal conditions, the maximum predicted value of H_max_ was 4703.23 mLL^−1^ (Fig. 3). The regression equation is represented graphically using three-dimensional response surface (Fig. 4) and two-dimensional contour plots (Fig. 5). The response surface of hydrogen production showed a clear optimum point located within the boundary range, and the interaction effect between independent variables was insignificant with the *P*-value > 0.05.

### Validation of the identified optimal conditions

The confirmation experiment was conducted to test if the optimum conditions determined by the statistical approach could be practically applied to produce H_max_. Triplicates of batch experiments under optimal conditions were conducted. The predicted H_max_ was 4703.23 mLL^−1^ at the optimum condition (initial pH of 8.98, WMP concentration of 44.75%, 4.49 gL^−1^ sodium acetate, and 1.15 gL^−1^ ammonium acetate) as suggested by BB design. The verification experiment revealed only a 0.59% difference between the predicted and experimental hydrogen production under optimal conditions.

**Fig. 6:**
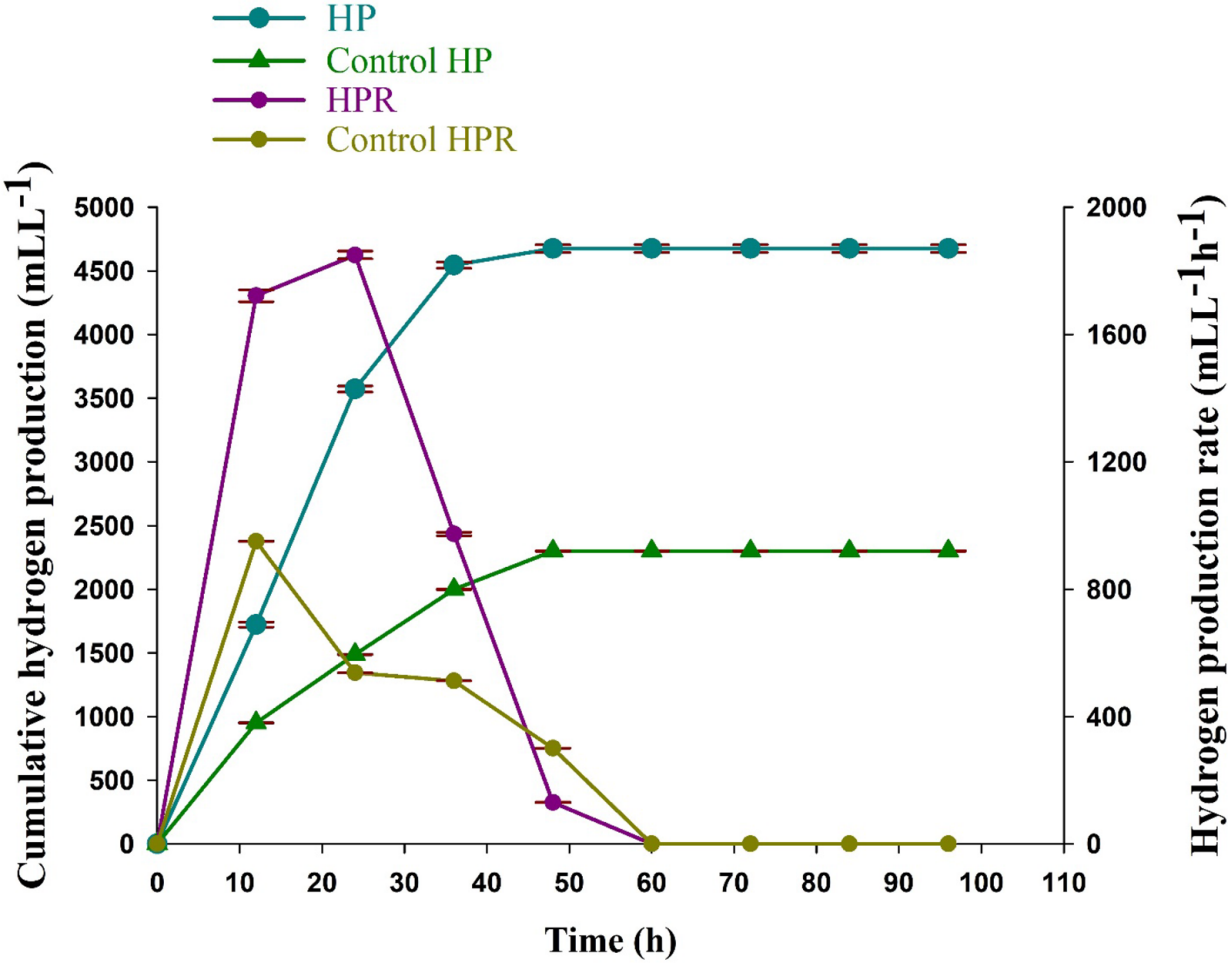
Hydrogen production and hydrogen production rate (HPR) from WMP at optimized conditions and without optimization (control)

The overall performance of hydrogen production under optimal conditions in 96 h is shown in Fig. 6. Results from the confirmation experiment calculated by the modified Gompertz equation showed H_max_ of 4675.33 ± 30.24 mLL^−1^, R_max_ (Experimental) of 1849.67 ± 12.32 mLL^−1^h^−1^, R_max_ (MGM) of 1721.37 mLL^−1^h^−1^, t_95_ of 25.55 h and R^2^ of 0.97273. Hydrogen production began after a short lag phase of 21.85 h and the hydrogen production rate maintained a high level at 12–24 h and then gradually decreased until it reached zero. On the other hand, kinetic analysis of WMP (before the optimization process) showed H_max_ of 2300.33 ± 1.15 mLL^−1^, R_max_ (Experimental) of 950.67 ± 0.88 mLL^−1^h^−1^, R_max_ (MGM) of 922.07 mLL^−1^h^−1^, short **λ** 22.36 h and t_95_ 25.89 h (Table 6). It was estimated that an enhancement of about 103.25% in hydrogen production by *C. butyricum* NE133 was achieved after the optimization process.

**Table 6:** Kinetic analysis of hydrogen production by *C. butyricum* NE133 at the optimum conditions using a modified Gompertz model

Conditions	Experimental		MGM			
	H_max_ (mLL^−1^)	R_max_ (mLL^−1^h^−1^)	λ (h)	R_max_ (mLL^−1^h^−1^)	t_95_ (h)	R^2^
Control^a^	2300.33 ± 1.15	950.67 ± 0.88	22.36	922.07	25.89	0.99992
Optimum^b^	4675.33 ± 30.24	1849.67 ± 12.32	21.85	1721.37	25.55	0.97273

a = WMP before the optimization process, b = WMP at the optimum condition (initial pH of 8.98, WMP concentration of 44.75%, 4.49 gL^−1^ sodium acetate, and 1.15 gL^−1^ ammonium acetate)

### Gas chromatography–mass spectrometry (GC–MS) analysis of the optimized WMP medium extract before and after fermentation

GC–MS analysis was conducted on the optimized WMP medium extract at the optimum conditions (initial pH of 8.98, WMP concentration of 44.75%, 4.49 gL^−1^ sodium acetate, and 1.15 gL^−1^ ammonium acetate) after fermentation and compared with the WMP control medium before fermentation. Obtained results of GC–MS analysis are shown in Table 7. The control medium (Fig. S3, supplementary file) contains fatty acids such as stearic acid (8.44%), linoleic acid (1.55%), palmitic acid (6.68%), bis(2-ethylhexyl) phthalate (32.51%), oleic acid (0.87%) and others. After the fermentation process of the optimized WMP medium, certain organic acids such as butyric acid, 4 hydroxy2 methylene (2.03%) and Z,Z3,15-octadecadien1-ol-acetate (1.05%) were detected (Fig. S4, supplementary file). Palmitic acid levels decreased and were detected at a percentage of 1.29%.

**Table 7:** Gas-chromatography analysis for WMP fermentation media extract before and after fermentation

Medium	Compound	Formula	Retention time	Area	Area (%)
Pretreated watermelon peels control medium	Bis(2-ethylhexyl) phthalate	C_24_H_38_O_4_	47.22	49,648,917.63	32.51
	9,12-Octadecadienoic acid (Z, Z) (Linoleic acid)	C_18_H_32_O_2_	40.91	2,366,643.10	1.55
	Octadecanoic acid (Stearic acid)	C_18_H_36_O_2_	37.75	12,892,066.34	8.44
	Hexadecanoic acid (Palmitic acid)	C_16_H_32_O_2_	41.31	10,207,942.17	6.68
	Hexadecadienoic acid, methyl ester	C_17_H_30_O_2_	41.07	1,923,123.81	1.26
	Oleic acid, eicosyl ester (CAS)	C_38_H_74_O_2_	49.34	1,334,310.29	0.87
	Hexadecatrienoic acid, methyl ester, labeled with carbon13(CAS)	C_17_H_28_O_2_	42.08	533,174.46	0.35
	Rhodoxanthin	C_40_H_50_O_2_	48.43	721,462.42	0.92
	Lucenin 2	C_27_H_30_O_16_	50.49	695,574.96	0.85
	Lycoxanthin	C_40_H_56_O	50.80	2,767,267.82	1.81
Optimized watermelon peels spent medium	Bis(2-ethylhexyl) phthalate	C_24_H_38_O_4_	47.29	14,612,282.90	21.79
	Palmitic acid	C_16_H_32_O_2_	41.41	867,259.05	1.29
	9Hexadecenoic acid, 9octadecenyl ester, (Z, Z) (CAS)	C_34_H_64_O_2_	51.51	1,366,743.69	2.04
	9Octadecenoic acid (Z), 9octadecenyl ester, (Z)(CAS)	C_36_H_68_O_2_	53.80	675,950.07	1.01
	Butyric acid, 4 hydroxy2 methylene	C_5_H_8_O_3_	49.90	1,360,464.37	2.03
	Z, Z3,15Octadecadien1-ol-acetate	C_20_H_36_O_2_	52.80	705,827.33	1.05

## Discussion

Hydrogen production via dark fermentation is greatly influenced by environmental factors such as temperature, pH, inoculum size, substrate concentration, and nutrient supplementation [23]. To maximize hydrogen production there is a need to optimize these environmental factors. In the current study, the significance of eight variables (initial pH, incubation temperature, WMP concentration, inoculum volume, yeast extract, tryptone, sodium acetate, and ammonium acetate concentration) was examined for hydrogen production by *C. butyricum* NE133 using a PB design. PB design is a valuable statistical tool used to evaluate the effects of variables on hydrogen production as it can significantly reduce the number of repeated experiments needed for a subsequent optimization study, using RSM [26, 27].

The results of PB design in the present study indicated that four variables including, initial pH, WMP concentration ammonium acetate, and sodium acetate were statistically significant (*P* < 0.05) for hydrogen production by *C. butyricum* NE133. Additionally, based on the main effects plot for hydrogen production in Fig. 1, it was observed that the high levels of pH, WMP concentration, and sodium acetate had the greatest positive impact on hydrogen production, respectively. Furthermore, it was reported that the initial pH value was a highly significant factor (*P* < 0.01) in controlling hydrogen production. An optimal pH level can enhance the activity of enzymes required to break down the substrate and produce hydrogen in dark fermentations thus improving the overall performance of the organism during fermentation [28]. This could be attributed to the production of volatile fatty acids during hydrogen production. If the initial pH did not inhibit bacterial growth, a higher initial pH value would have the advantage of delaying the onset of pH inhibition during hydrogen production caused by the metabolic shift from acidogenesis to solventogenesis [29].

The results also showed that the substrate concentration (WMP concentration) significantly affects the yield of H_2_. The production of products is closely related to the availability of the substrate. Therefore, if the concentration is not adjusted, problems such as mass transfer limitations, accumulation of organic acids, and inhibition of hydrogen-producing microorganisms may occur, preventing maximum hydrogen production. A high substrate concentration becomes inhibitory to the microorganisms as a result of a pH drop or hydrogen partial pressure increase. Conversely, at low substrate concentrations bacteria are thought to utilize the carbon source mainly for biomass growth and not biogas production [30].

The addition of sodium acetate to the fermentation medium had a considerable impact on the cumulative hydrogen production by *C. butyricum* NE133. This might be because acetate is a buffering supplement that can regulate pH levels and mitigate medium acidity. Acetate can effectively control pH changes during fermentative hydrogen production, increasing hydrogen yield [31]. They demonstrated that increasing the concentration of sodium acetate to 50 mM can boost the hydrogen yield. However, when the concentration of acetate exceeded 50 mM, there was a slight inhibition of hydrogen production.

The efficiency of the fermentation process is significantly affected by the medium composition, including carbon, nitrogen, vitamins, and minerals [32]. Nitrogen source is necessary for metabolism, cell division, maintenance, and the synthesis of enzymes that break down carbohydrates, it also influences the organism’s activity [33]. The present study investigated three factors: ammonium acetate, tryptone, and yeast extract as a nitrogen source for fermentative hydrogen production by *C. butyricum* NE133. Relying on the main effects plot for hydrogen production in Fig. 1, it could be concluded that ammonium acetate showed a significant negative impact on hydrogen production. Higher concentrations of ammonia can decrease the hydrogen production potential [34–36]. Moreover, the results also indicated that the high level of tryptone, and yeast extract had a negative insignificant effect on the fermentation process. These findings are similar to those of Karthic et al. [37] who screened the effect of factors such as initial pH, inoculum size, glucose, yeast extract, tryptone, and ferric chloride on hydrogen production by *Enterobacter aerogenes* MTCC 111 using PB design. They reported that inoculum size, yeast extract, and tryptone had a negative insignificant (confidence levels below 95%) effect on hydrogen production, while initial pH, glucose, and ferric chloride had a positive significant (confidence levels above 95%) effect.

In many microbial systems, the size of the inoculum can have an impact on product formation and growth. The obtained results indicated that the high level of inoculum volume ratio (IVR) showed an insignificant positive effect on hydrogen production. Similar results were revealed by Zhang et al. [38], a 20% IVR resulted in the highest hydrogen yield (85.6 mL/g TS), and this IVR did not show a significant difference (*P* > 0.05) from other IVRs. Moreover, they observed that hydrogen yield decreased to 62.5 mL/g TS when the IVR was raised to 45%. This suggested that very high IVR levels may impede hydrogen production by competing with cell mass and reducing light conversion efficiency due to flocculation. Nonetheless, a much lower hydrogen production was achieved with a lower IVR level, indicating that insufficient bacterial supplementation caused the lower yield. Moreover, the obtained results in Fig. 1 showed that a high level of temperature had a negative insignificant effect on hydrogen production by *C. butyricum* NE133. In DF, temperature influences the thermodynamic equilibrium of biochemical reactions and affects the activity of hydrogen-producing bacteria, which in turn influences the yield and rate of hydrogen production. Therefore, choosing a suitable operating temperature for the fermentation process is necessary. Marone et al. [39] studied the self-fermentation of some cellulosic substrates to produce hydrogen under two anaerobic conditions (28 °C and 37 °C). They revealed that the highest hydrogen production and hydrogen production rates were obtained at the lower temperature, close to ambient temperature (28 °C). Also, Gadhamshetty et al. [40] reported that hydrogen production was 30% higher in a reactor operating at 22 °C compared to 37 °C. They attributed these results to gradual pH changes caused by slower kinetics at lower temperatures. The reason might be that lower temperatures provide more time for hydrogen-producing bacteria to adapt to pH dynamics in unbuffered reactors. Infantes et al. [41] found that 26 °C always exhibited the highest cumulative hydrogen production, independent of the pH. These findings were in agreement with ours, confirming that the hydrogen yield of the fermentation process is higher at lower temperatures.

Analyzing the curvature of the first-order model (PB design) gave a significant difference (*P* = 0.040), indicating that the responses are located in the optimal region and can be optimized in the next optimization step [42]. To maximize and approach the optimum response region of hydrogen production, the significant independent variables; initial pH(X_1_), WMP concentration(X_4_), sodium acetate (X_5_), and ammonium acetate (X_6_) suggested by the Plackett–Burman design were further investigated by Box–Behnken design [24]. Sun et al. [16] revealed that Placket–Burman and Box–Behnken designs have been used to optimize process parameters of biohydrogen production by pure strains [43]. This approach was preferably selected to reduce the cost of energy and chemicals in the experiments. In BB design, all tested variables are investigated at their low and high levels at the same time. As a result, this design is useful to avoid unsatisfactory results caused by extreme conditions [44]. The high R^2^ coefficient of our results (0.9902) indicated that the quadratic model properly fits the experimental results. BB design was utilized to optimize the concentrations of chosen variables for maximizing hydrogen production and to investigate the interactive effects between the tested factors [45, 46]. In the current study, the obtained results showed that initial pH, WMP concentration, and sodium acetate concentration all had an individual significant effect on HP from watermelon peels. The optimal conditions for maximizing hydrogen production from WMP by *C. butyricum* using Box–Behnken design were: initial pH of 8.98, WMP concentration of 44.75%, sodium acetate 4.49 gL^−1^, and ammonium acetate 1.15 gL^−1^. Similar findings were reported by Camargo et al. [13] who revealed that the highest values of hydrogen production were obtained at a pH of 8.98 for the optimized condition.

A confirmation experiment was carried out to validate the accuracy of optimum conditions determined by the statistical approach for maximum hydrogen production. By comparing the experimental results with the predicted value of hydrogen production under optimum conditions, the difference was only 0.59%. Therefore, the optimal conditions estimated by BB design for HP from WMP through dark fermentation had the least error and could be practically applied. Also, kinetic analysis using MGM for hydrogen production under these optimal conditions confirmed the statistical design. The modified Gompertz model (MGM) has been widely used for sustainable biohydrogen production through dark fermentation [25]. Basak et al. [47] used the modified Gompertz model (MGM) and logistic model to study the kinetics of biohydrogen production by pretreated anaerobic sludge through dark fermentation of FVW and cottage cheese whey and to define the kinetic parameters, which helped in the upscaling of HP processes. MGM was selected because it is the most frequently used kinetic model applied for simulating dark fermentation [48]. It has been widely employed to assess the fermentation processes due to its simplicity, ease of application and lower data need [49].

WMP is considered an applicable natural substrate for microbial fermentation to produce hydrogen. The high content of reducing sugars and availability of the essential minerals in WMP facilitates the efficient microbial fermentation, and supports the metabolic pathways and enzymatic reactions critical for hydrogen production [18]. GC–MS analysis of the WMP fermentation media extract was performed before fermentation by *C. butyricum* NE133. Fatty acids like stearic acid, palmitic acid, linoleic acid and oleic acid were recorded, which was consistent with the previous studies of Petchsomrit et al. [50]. Certain organic acids, such as butyric acid and acetic acid were recorded after fermentation. Dionisi and Silva [51] indicated that hydrogen is produced when butyric acid and acetic acid are produced. Furthermore, some fatty acids in WMP such as linoleic acid and palmitic acid could help to enhance hydrogen production, by inhibiting hydrogen-consuming microbes and redirecting electron equivalent to HP [52, 53]. Also, palmitic acid was the best-performing fatty acid in enhancing hydrogen yield [54]. This indicates the importance of using WMP as a substrate for *C. butyricum* NE133 under these optimized conditions. Using this optimization strategy, hydrogen production increased significantly from 2300.33 to 4675.33 mLL^−1^, demonstrating the high hydrogen-producing capability of the microorganism used in this study under optimal conditions.

## Conclusion

The findings presented in the study indicated the successful use of statistical optimization to improve hydrogen production from watermelon peels (WMP) by *Clostridium butyricum* NE133. Using Plackett–Burman (PB) and Box–Behnken (BB) designs, it was established that four factors (initial pH, WMP concentration, ammonium acetate, and sodium acetate) were positively correlated (*P* < 0.05) with hydrogen production. Model accuracy was validated by the high R^2^ and adjusted R^2^, which was confirmed by kinetic analysis using the MGM. Experimental validation yielded a slight 0.59% deviation from the predicted yield, further demonstrating the reliability and real applicability of the optimized conditions. Furthermore, hydrogen production was improved by 103.25% after optimizing with a maximum hydrogen production of 4675.33 ± 30.24 mL/L. This significant increase demonstrated the effectiveness of our optimization strategy and positions our study among the highest yields reported for hydrogen production from fruit peels, indicating the potential of WMP as a promising sustainable source. Future research can be aimed at developing a larger scale for industrial applications and uncovering the specific metabolic pathways of *C. butyricum* NE133 to further enhance hydrogen production efficiency.

## Supplementary Information

Additional file 1: Table S1. Physicochemical composition of WMP. **Fig. S2.** A designed hydrogen production system using water displacement method. **Fig. S3.** GC–MS analysis of pretreated WMP control medium before fermentation. **Fig. S4.** GC–MS analysis of optimized WMP spent medium after fermentation

## Data Availability

No datasets were generated or analysed during the current study.

## Abbreviations

DF: Dark fermentation
PB: Plackett–Burman
RSM: Response surface methodology
BB: Box–Behnken
WMP: Watermelon peel
GC–MS: Gas chromatography–mass spectrometry
FAE: Fatty acid ester
VFAs: Volatile fatty acids
X_i_: Independent variable
E_Xi_: Effect of the tested variable
MGM: Modified Gompertz model
H_max_: Maximum hydrogen production
R_max_: Maximum hydrogen production rate
λ: Lag phase
(H): Cumulative hydrogen production
H(c): Hydrogen production potential
HPR: Hydrogen production rate
