# Supplementary material for: Maximizing biohydrogen production from watermelon peels using Clostridium butyricum NE133: a statistical optimization approach with Plackett–Burman and Box–Behnken designs
Source: Biotechnol Biofuels Bioprod. 2025 May 14;18:54. doi: 10.1186/s13068-025-02652-3 (PMC12079854; doi:10.1186/s13068-025-02652-3)
Supplement: Supplementary file 1 — Additional file 1: Table S1. Physicochemical composition of WMP. Fig. S2. A designed hydrogen production system using water displacement method. Fig. S3. GC–MS analysis of pretreated WMP control medium before fermentation. Fig. S4. GC–MS analysis of optimized WMP spent medium after fermentation [file 13068_2025_2652_MOESM1_ESM.docx]

**Table S1.** Physicochemical composition of WMP

| Parameters | Concentration (per 100 g) |
| --- | --- |
| COD (mg) | 3695.66±13.03 |
| TDS) mg) | 6376±17.24 |
| Reducing sugars (g) | 48.5±0.27 |
| Total protein) g) | 6.11±0.18 |
| Total ash (g) | 13.44±0.16 |
| Moisture (%) | 10.15±0.23 |
| Na) g) | 6.60±0.23 |
| K (g) | 2.113±0.17 |
| Mg) g) | 0.201±0.09 |
| Ca) g) | 1.63±0.15 |
| pH | 6.26 |

**Fermentation medium**


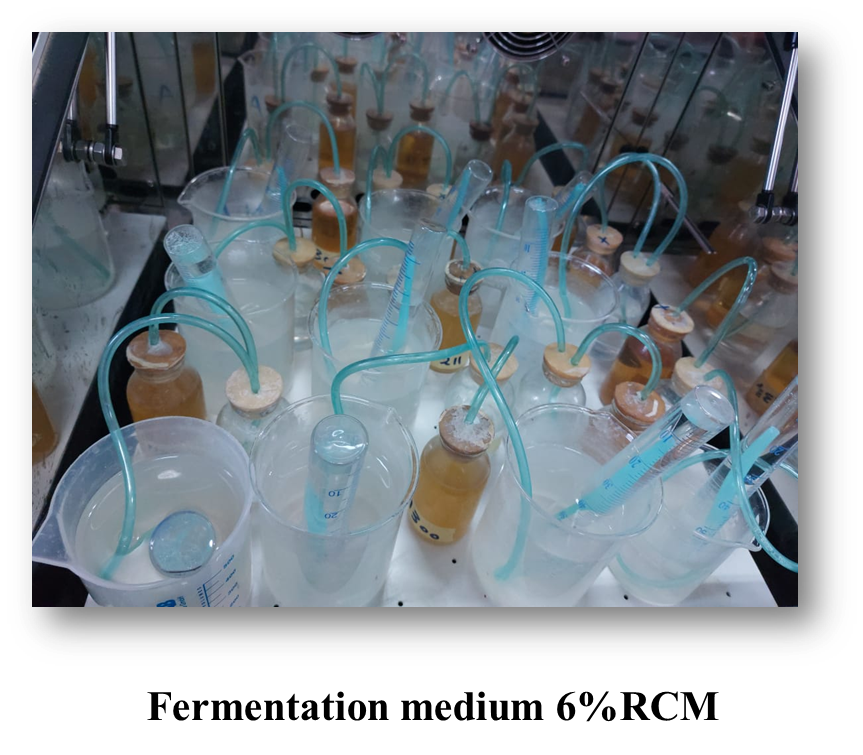


**2M NaoH**

**Cumulative hydrogen gas**

**Fig. S2.** A designed hydrogen production system using water displacement method.


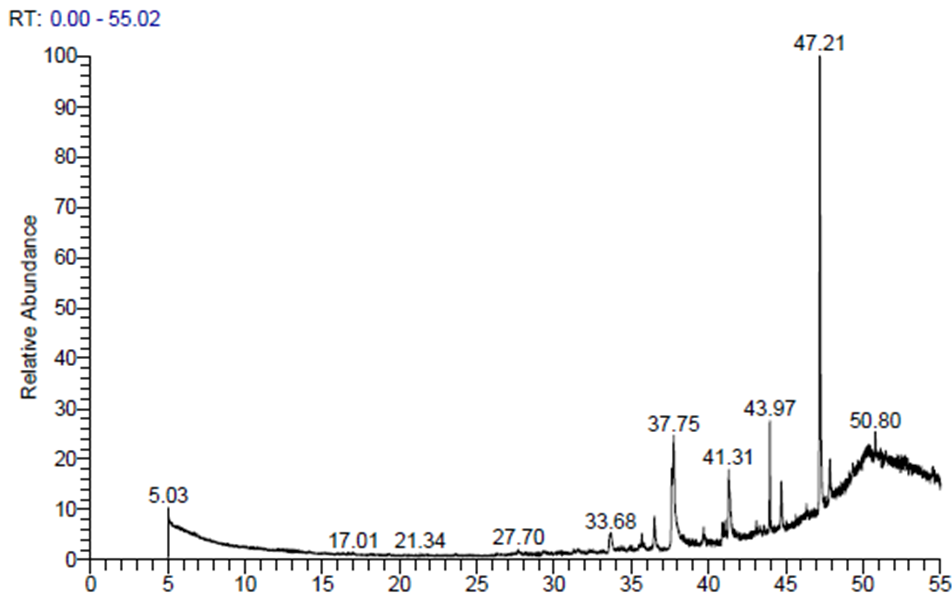


**Fig. S3.** GC-MS analysis of pretreated WMP control medium before fermentation


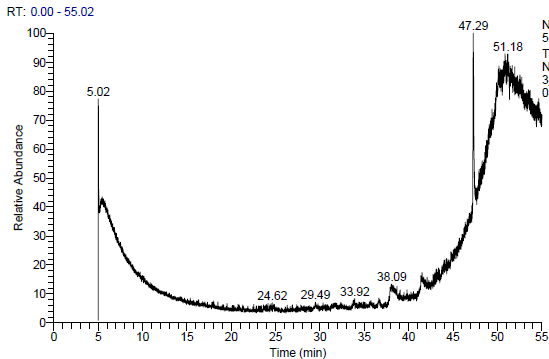


**Fig. S4.** GC-MS analysis of optimized WMP spent medium after fermentation
